# Systematic Review and Exploratory Meta‐Analysis of AI‐Enabled and Digital Technology‐Assisted Interventions for Dental Anxiety During Dental Treatment

**DOI:** 10.1155/da/8850179

**Published:** 2026-07-02

**Authors:** Mohammad Ali Saghiri, Ravinder S. Saini, Ahid Amer Alshahrani, Sunil Kumar Vaddamanu, Ali Alqahtani, Nadim Z. Baba

**Affiliations:** ^1^ Department of Restorative Dentistry, Director of Biomaterial and Prosthodontic Laboratory, Rutgers School of Dental Medicine, Newark, New Jersey, USA, rutgers.edu; ^2^ Department of Allied Dental Health Sciences COAMS, King Khalid University, Abha, Saudi Arabia, kku.edu.sa; ^3^ Department of Periodontics and Community Dentistry, King Khalid University, Abha, Saudi Arabia, kku.edu.sa; ^4^ Advanced Specialty Education Program in Implant Dentistry, School of Dentistry, Loma Linda University, California, USA, llu.edu

## Abstract

**Background:**

Dental anxiety adversely affects receiving dental care when needed. Artificial intelligence (AI)‐enabled and digital technology‐assisted interventions have been recently explored for assessment, monitoring or alleviation of anxiety during dental treatment. The aim of this systematic review was to summarise current evidence and review effectiveness of AI‐enabled or digital technology‐assisted interventions on anxiety and treatment‐related outcomes in dentistry.

**Method:**

The review was conducted in accordance with Preferred Reporting for Systematic Reviews and Meta‐analysis (PRISMA) guidelines. Searches were conducted across ScienceDirect, PubMed, Scopus, Google Scholar and Cochrane Library. There were no date limitations applied. Search was restricted to human studies involving AI‐enabled or digital technology‐assisted interventions for dental anxiety management. Both interventional and observational studies were included for review. Data were extracted regarding study characteristics, intervention type, anxiety outcome measures, behavioural response, treatment adherence and satisfaction. Risk of bias was assessed utilising Mixed Methods Appraisal Tool (MMAT). Owing to significant clinical and methodological heterogeneity among the included studies, quantitative synthesis was limited to a small subset of comparable studies and was therefore considered exploratory.

**Results:**

Seven studies met our eligibility criteria. Eligible studies varied widely in design, population, intervention and outcome assessment modalities used. These were computerised cognitive behavioural therapy (CBT), humanoid robots, smartphone applications, machine learning (ML) approaches to assessment, AI‐linked virtual reality (VR) or biofeedback programs. Narrative synthesis suggested that several interventions were associated with reductions in dental anxiety or improvements in patient behaviour. Quantitative pooling was possible for only a few studies which were found to be comparable. Our conclusions from this exploratory meta‐analysis are limited by the small number of studies with comparison groups available and considerable heterogeneity.

**Conclusion:**

AI‐enabled and digital technology‐assisted interventions have potential to improve dental anxiety‐related outcomes and dental patient experience. The evidence is currently limited. The available studies are small and heterogeneous and methodologically limited. Future investigations should include larger, robust trials with standardised outcome measures and consistent operational definitions of AI.

## 1. Introduction

Dental anxiety or fear has been accepted as one of the common causes for seeking late dental care [1, 2]. Dental anxiety can lead to missed appointments, delayed treatment, and disease progression. This can lead to poor oral‐health‐related outcomes. Several reasons have been attributed to dental anxiety [3, 4]. These include previous negative dental experiences, fear of pain/discomfort, loss of control, fear of negative expectations and overall psychological vulnerability [5, 6]. Manifestations of dental anxiety include physiological arousal, psychological distress, behavioural resistance, and non‐cooperation during dental treatment among both adults and children [7, 8]. Anxiety may result in poorer adherence to treatment. Timely detection and management of dental anxiety can be critical to providing patient‐centred dental care [9, 10].

Behavioural instruction, distraction, relaxation training, cognitive behavioural methods, sedation and pharmacological techniques have all been employed. All these techniques are used in efforts to prevent and treat dental anxiety. Many of these interventions may be constrained by availability, costs, variable acceptability to patients and differing effectiveness among patients and clinical situations [11, 12]. Artificial intelligence (AI)‐enabled and digital technology‐assisted systems have recently been introduced as methods to aid in anxiety assessment, prediction, monitoring and behaviour management [13, 14]. Examples have included computerised therapeutic interventions, smartphone applications, virtual reality (VR)‐connected tools, biofeedback‐informed platforms, robotics and machine learning (ML)‐based assessment tools. The present body of literature is conceptually and methodologically diverse and it is often unclear if the implemented technology was used to assess and address anxiety through AI interventions, digital tools, or a combination of both. This important consideration will likely affect how this evidence can be interpreted and implemented as well as how future studies are designed [15, 16].

To date, no review has summarised the evidence surrounding AI‐enabled interventions as well as other digital tools used specifically for dental anxiety and their effects on treatment‐related outcomes. The current literature evaluates mixed study designs, consists of multiple age groups and uses varied formats for intervention delivery. It employs a variety of anxiety outcome measures. We aimed to conduct a systematic review of AI‐ and digital technology‐assisted interventions for dental anxiety.

The objective of this systematic review was to determine the available evidence on AI‐enabled and digital technology‐assisted tools specifically designed or applied to measure, monitor, predict or alleviate dental anxiety in paediatric or adult patients receiving dental treatment. Secondary objectives were to help discern the use of true AI‐enabled methods vs. the broader use of digital technology‐assisted interventions. It was also to evaluate whether these tools impacted treatment‐related outcomes such as behavioural response compliance, physiological anxiety response and patient experience. Our research question was: In patients receiving dental care, do AI‐enabled or digital technology‐assisted tools specifically applied to measure or manage dental anxiety improve anxiety scores or treatment‐related outcomes compared with baseline, standard care, or non‐AI comparators?

We operationally defined AI‐enabled intervention as any tools or systems that utilised ML, automated prediction, automated adaptive decision‐making, intelligent classification or automated feedback loops for the assessment, monitoring, prediction or management of dental anxiety. Digital technology‐assisted interventions were defined as any digital tools that were not AI‐driven such as computerised cognitive behavioural therapy (CBT) programs, smartphone applications, VR systems, robots or scales utilised specifically for assessment, behaviour management or anxiety reduction related to dental anxiety. Digital technology utilised for any purpose within dentistry did not make the study eligible for review unless the technology was clearly stated to be used for assessment, monitoring, prediction or reduction of dental anxiety.

## 2. Methodology

### 2.1. Study Design

The present study had a well‐defined search process starting from identification to inclusion of studies, followed by the evaluation of the most relevant literature in terms of the protocols provided by Preferred Reporting for Systematic Reviews and Meta‐analysis (PRISMA) to ensure replication and transparency of the studies [17]. The protocol for this systematic review was registered on the International Platform of Registered Systematic Review and Meta‐Analysis Protocols (INPLASY) (Registration Number: 2024120055). The existing research presents diverse interventions, study structures and ways of measuring anxiety. We opted for a systematic review as our main approach, incorporating a limited quantitative synthesis when relevant.

### 2.2. Research Question

In patients receiving dental care, do AI‐enabled or digital technology‐assisted interventions assess, monitor, reduce dental anxiety and improve treatment outcomes when compared with baseline or a non‐AI comparator?

### 2.3. PICO Framework

The review question was created in PICO format: Population (P): children and adults undergoing dental treatment or dental anxiety assessment in dental clinics. Intervention (I): AI‐enabled or digital technology‐assisted tools used specifically for dental anxiety measurement, prediction, tracking, behaviour management or anxiety alleviation. AI‐enabled tools were those utilising ML, automated risk assessment, adaptive decision‐making, smart classifications or algorithmic feedback loops. Digital technology‐assisted tools were defined as computerised CBT (C‐CBT), smartphone applications, VR systems, robot‐assisted tools or digital scales when the tool was employed specifically for dental anxiety and not solely for general dentistry. Comparator (C): standard care/no intervention/wait‐list control/non‐AI comparator/pre‐intervention baseline. Outcomes (O): dental anxiety scores, behavioural outcomes, physiological measures of anxiety, treatment compliance and patient experience.

### 2.4. Search Strategy

An advanced literature search was performed using different databases, including ScienceDirect, PubMed, Scopus, Google Scholar and the Cochrane Library, without date restrictions. The search strategy was structured in accordance with the PICO framework and combined intervention‐related terms such as ‘artificial intelligence’, ‘AI‐enabled’, ‘machine learning’, ‘deep learning’, ‘robotics’, ‘virtual reality’, ‘biofeedback’ and ‘mobile applications’ with outcome‐related terms such as ‘dental anxiety’, ‘dental fear’ and ‘dental phobia’ using Boolean operators (AND, OR) adapted for each database. Full database‐specific search strings are provided in Table 1 and Supporting Information Table S1.

**Table 1:** Literature search from different databases.

Database	Search terms
	(‘Artificial intelligence interventions’ [Title/Abstract] OR ‘AI‐based interventions’ [Title/Abstract] OR ‘Virtual reality’ [MeSH Terms] OR ‘Biofeedback’ [Title/Abstract] OR ‘Deep learning’ [Title/Abstract] OR ‘Neural networks’ [Title/Abstract] OR ‘Natural language processing’ [MeSH Terms] OR ‘Robotic intervention’ [Title/Abstract] OR ‘Virtual assistants’ [Title/Abstract] OR ‘AI‐based system’ [Title/Abstract]) AND (‘Dental anxiety’ [Title/Abstract] OR ‘Dental fear’ [Title/Abstract] OR ‘Dental phobia’ [Title/Abstract])
The Cochrane Library	((‘Artificial intelligence interventions’ OR ‘AI‐based interventions‘ OR ‘Virtual reality’ OR ‘Biofeedback’ OR ‘Deep learning’ OR ‘Neural networks’ OR ‘Natural language processing’ OR ‘Robotic intervention’ OR ‘Virtual assistants’ OR ‘AI‐based system’)):ti,ab,kw AND ((‘Dental anxiety’ OR ‘Dental fear’ OR ‘Dental phobia’)):ti,ab,kw
ScienceDirect	(‘Artificial intelligence interventions’ OR ‘AI‐based interventions’ OR ‘Virtual reality’ OR ‘Deep learning’ OR ‘Natural language processing’ OR ‘AI‐based system’) AND (‘Dental anxiety’ OR ‘Dental fear’ OR ‘Dental phobia’)
Scopus	(‘Artificial intelligence interventions’ OR ‘AI‐based interventions’ OR ‘Virtual reality’ OR ‘Biofeedback’ OR ‘Deep learning’ OR ‘Neural networks’ OR ‘Natural language processing’ OR ‘Robotic intervention’ OR ‘Virtual assistants’ OR ‘AI‐based system’) AND (‘Dental anxiety’ OR ‘Dental fear’ OR ‘Dental phobia’)
Google Scholar	(‘Artificial intelligence interventions’ OR ‘AI‐based interventions’ OR ‘Virtual reality’ OR ‘Deep learning’ OR ‘Natural language processing’ OR ‘AI‐based system’) AND (‘Dental anxiety’ OR ‘Dental fear’ OR ‘Dental phobia’)

### 2.5. Eligibility Criteria

Studies were included if they reported original research evaluating an AI‐enabled tool or digital technology‐assisted tool that was explicitly used to assess, monitor, predict and manage behaviour related to reduction of anxiety during dental care. AI‐enabled tools included systems that used ML, automated prediction, intelligent classifications, adaptive decision‐making or algorithmic feedback. Digital technology‐assisted tools were included only if they were used explicitly to assess, monitor or reduce dental anxiety or behaviour related to dental anxiety. We excluded studies that did not specifically apply digital technology or AI to address dental anxiety just because the technology was used in dental settings or because they used AI‐related terms without any specific application to address dental anxiety or measured outcomes not related to dental anxiety. Included studies used human subjects and reported original data with measurable dental anxiety‐related or dental treatment‐related outcomes. Studies were included if they were randomised controlled trials, non‐randomised interventional studies, observational studies, and validation studies. We excluded reviews, editorials, non‐peer reviewed documents, studies that did not involve human subjects, and studies that used digital tools for purposes other than addressing dental anxiety.

### 2.6. Study Selection

The title and abstracts of all retrieved articles were screened independently by two reviewers. Duplicate articles were removed with EndNote X9, and disagreements were resolved manually. Full‐text articles were assessed by two reviewers against the inclusion/exclusion criteria. Articles sourced from grey literature searches and non‐peer reviewed documents were excluded. Any disagreement between reviewers was discussed and settled with the consultation of a third reviewer if necessary.

### 2.7. Data Extraction

Predefined data variables were extracted using Microsoft Excel. The included variables were study characteristics (study ID, country, study design and sample size), participant characteristics (age, sex and anxiety symptoms), intervention characteristics (type of AI intervention, delivery mode, duration, frequency and control group), and outcomes (anxiety reduction, treatment adherence, physiological response, pain perception, follow‐up, adverse events, key findings and conclusion). Data were extracted to be used for the narrative synthesis of all included studies and where appropriate for the limited exploratory quantitative synthesis of comparable studies.

### 2.8. Quality Assessment

Quality was evaluated using the Mixed Methods Appraisal Tool (MMAT) quality scoring system to determine how well the methodology of each study conformed to pre‐established standards. (Table 2) [18, 19].

**Table 2:** MMAT quality assessment items.

Items for RCTs
2.1. Is randomisation appropriately performed?
2.2. Are the groups comparable at baseline?
2.3. Are there complete outcome data?
2.4. Are outcome assessors blinded to the intervention provided?
2.5 Did the participants adhere to the assigned intervention?

**Items for non-RCTs**

3.1. Are the participants representative of the target population?
3.2. Are measurements appropriate regarding both the outcome and intervention (or exposure)?
3.3. Are there complete outcome data?
3.4. Are the confounders accounted for in the design and analysis?

MMAT was selected because the body of evidence contained randomised and non‐randomised designs of evidence. Studies were evaluated according to the MMAT criteria checklist specific to that study’s design. We utilised the quality ratings to guide our interpretation of the findings and to identify methodological weaknesses, without using them as a strict cutoff for inclusion.

### 2.9. Statistical Analysis

Tables and figures were prepared using Microsoft Excel. An exploratory quantitative synthesis was performed using RevMan 5.4 (random‐effects model) for all outcomes. There was considerable heterogeneity between studies about study design, types of interventions and anxiety outcome measures. Due to this, our quantitative results are interpreted cautiously. Statistical heterogeneity was explored using the Cochran’s *Q* test and *I* ^2^ statistic. Tests for evaluation of publication bias and meta‐regression were not considered, as there were a low number of studies that were eligible for quantitative synthesis.

## 3. Results

### 3.1. Literature Searched

**Figure 1:**
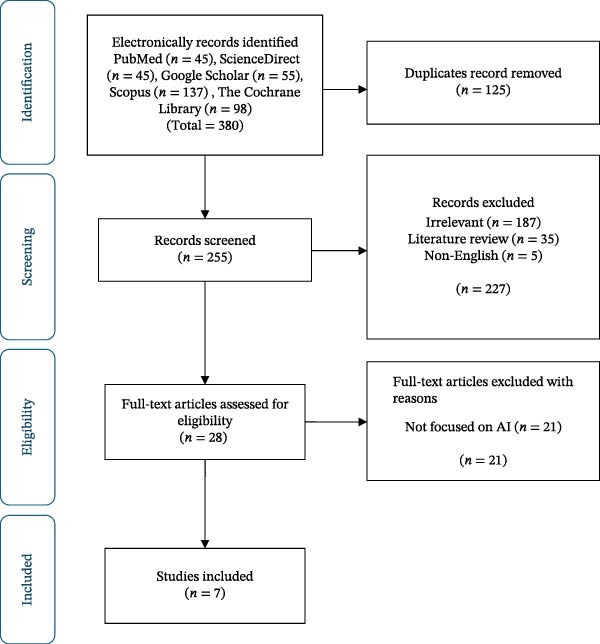
PRISMA flow chart for study selection.

Initially, 380 research articles were retrieved from different databases, including PubMed, ScienceDirect, Google Scholar, the Cochrane Library and Scopus. After retrieval, 125 research articles were duplicated and removed before starting the title and abstract screening processes. Subsequently, 255 research articles were evaluated for eligibility, and the selection criteria were strictly followed. After thorough and comprehensive screening, 227 research articles were not found in accordance with the aim of the study and inclusion criteria and were excluded. After screening, only 28 research articles were eligible for the full‐text assessment. Among these, 21 research articles were excluded for the reasons given in Figure 1. Finally, seven articles were included in the present review.

### 3.2. General Characteristics

Table 3 presents the general characteristics of seven studies, reflecting diverse geographical regions, including the United States of America (USA) [21], Sweden [20], Turkey [22], China [24], Pakistan [23], Saudi Arabia [25], and the United Arab Emirates (UAE) [26], and study designs such as retrospective [20], cross‐sectional [25, 26], RCTs [21, 23], and participatory [24]. Most of the studies included children [20, 22, 23, 26], and a few studies targeted adults [21, 25], while a single study covered both children and adults [24], with sample sizes ranging from 50 to 3204 participants [20, 25]. The sex distribution varied, and male‐to‐female ratios were provided in each study. Notably, most studies focused on patient populations, except for one study that also included medical staff, with a few targeting healthy or mentally ill participants [24, 25].

**Table 3:** Summary of the general characteristics of the included studies.

Study ID	Country	Study design	Sample size	Age (years)	Gender (M:F)	Stage	Population	Disability
Klingberg et al. [20]	Sweden	R	3204	4–11	1550:1654	Children	Patients	NA
Tellez et al. [21]	USA	RCT	151	44.7	58:93	Adult	Patients	NA
Kasimoglu et al. [22]	Turkey	Comparative	200	6.5	98:102	Children	Patients	Healthy
Abbasi et al. [23]	Pakistan	RCT	80	6.8	43:37	Children	Patients	Healthy
Huang et al. [24]	China	Participatory	180	3–74	76:104	Children and adults	Medical staff and patients	NA
Al Kheraif et al. [25]	Saudi Arabia	C	50	NA	0:90	Adult	Patients	Mental
Shetty et al. [26]	UAE	C	140	7.36	64:76	Children	Patients	Healthy

Abbreviations: C, cross‐sectional; F, female; M, male; NA, not available; R, retrospective; UAE, United Arab Emirates; USA, United States of America.

### 3.3. Characteristics of Included Interventions

These studies utilised different AI technologies, such as mobile apps [23, 24], ML [20], C‐CBT [21], humanoid robots [22] and AI‐based VR [25]. The delivery modes range from robots and mobile apps to computer‐based interventions. The duration and frequency of interventions varied, with some involving 1 h or short durations (e.g., 15–30 min) [21, 25] with one or a single session [22, 25]. Comparator conditions differed as well and consisted of wait‐list control groups, no treatment controls, therapy without robotics and baseline (i.e., before intervention) measures. Assessment of anxiety also differed across studies and consisted of Modified Dental Anxiety Scale (MDAS), Facial Image Scale (FIS), Frankl Behaviour Rating Scale (FBRS), GSR‐based measurements, Venham anxiety scale, Venham behaviour scale, CFSS‐DS, RMS‐DAS, RMS‐PS and others that were self‐reported by patients (Table 4).

**Table 4:** Summary of AI‐enabled and digital technology‐assisted tool characteristics used for dental anxiety assessment and management.

Study ID	Technology/tool type	Technology classification and dental anxiety relevance	Delivery mode	Duration	Frequency	Control group	Scale used for anxiety assessment
Klingberg et al. [20]	Inductive analysis program; machine learning	AI‐enabled; machine learning applied to dental fear and behaviour‐management problem assessment/prediction.	Not applicable	Not available	Not available	Not applicable	CFSS‐DS; BMP
Tellez et al. [21]	Computerised CBT dental anxiety intervention	Digital technology‐assisted; computerised CBT tool designed to reduce dental anxiety and avoidance.	Computer with headphone	1 h	Not available	Wait‐list control	CSQ‐8; MDAS
Kasimoglu et al. [22]	Humanoid robot	AI‐assisted/digital robotic tool; humanoid robot used to reduce child dental anxiety and improve behaviour.	Robot	Not available	1 session	Without robot	Parental Corah Dental Anxiety Scale; FIS; FBRS
Abbasi et al. [23]	Little lovely dentist mobile application	Digital technology‐assisted; mobile application used to reduce paediatric dental anxiety.	Mobile application	Not available	Not available	No treatment	FIS
Huang et al. [24]	Smartphone WeChat applet	Digital technology‐assisted; WeChat applet used for dental anxiety assessment and preoperative evaluation.	Mobile application	Not available	Not available	Not applicable	SUS
Al Kheraif et al. [25]	AI‐powered environment linked to GSR and VR	AI‐linked VR/biofeedback‐assisted tool; VR/AI environment used to reduce anxiety and improve behaviour.	Virtual reality with GSR‐linked environment	15–30 min	Single session	No treatment	GSR anxiety scores; FBRS; Venham anxiety and behaviour scale
Shetty et al. [26]	RMS scales mobile application	AI‐enabled digital assessment scale; AI‐based scale used for dental anxiety assessment in children.	Mobile application	Not available	Not available	Not applicable	RMS‐DAS; RMS‐PS; FIS

*Note:* CFSS‐DS, subscale of children’s fear survey schedule.

Abbreviations: BMP, behaviour management problems; CSQ‐8, client satisfaction questionnaire‐8; DAS, digital anxiety scale; FBRS, Frankl Behaviour Rating Scale; FIS, Facial Image Scale; GSR, galvanic skin response; MDAS, Modified Dental Anxiety Scale; NA, not available; PS, pictorial scale; SUS, system usability scale.

### 3.4. Outcomes

Table 5 summarises the outcomes of various AI‐based interventions and overall, AI‐based interventions across different platforms have been effective in reducing dental anxiety, enhancing treatment adherence and improving psychological responses. Findings were heterogeneous and should be interpreted cautiously; for instance, a C‐CBT tool significantly reduces dental anxiety (MDAS scores) and avoidance behaviours [21]. Similarly, robotic technology effectively reduces anxiety and improves behaviour in children during dental treatment [22]. Moreover, mobile applications lowered heart rates and anxiety, and the WeChat mobile app was reliable in reducing dental anxiety over 12 months [23, 24]. Similarly, VR combined with AI significantly reduced anxiety in patients with mental disabilities [25], and the RMS‐DAS is a reliable scale for assessing dental anxiety in children [26]. Many studies were based on assessment, prediction, usability or validation studies of anxiety scales instead of intervention studies. Therefore, the existing evidence can only be considered preliminary and heterogeneous.

**Table 5:** Summary of outcomes of AI‐enabled and digital technology‐assisted tools for dental anxiety assessment and management.

Study ID	Anxiety reduction	Treatment adherence	Psychological response	Pain/patient perception	Follow‐up	Key findings	Conclusion
Klingberg et al. [20]	NA	NA	NA	NA	NA	The knowledge trees of the two outcomes— dental fear and BMP—varied radically from one another	Dental BMP were more connected to dental variables
Tellez et al. [21]	MDAS: pre = 19.5, post = 15.4; ADIS‐IV: pre = 5.6, post = 3	Dental avoidance: pre = 4.6, post = 1.3	NA	Satisfaction rate = 83%	1 month	C‐CBT was found effective	It seems to be efficacious in reducing dental anxiety
Kasimoglu et al. [22]	FIS: pre = 1.84, post = 1.71; pulse rate: pre = 99.38, post = 97.03; FBRS: RG = 46%, CG = 15%	NA	Good	Satisfaction rate = 88.3%	NA	Dental treatment resulted in significantly (*p*0.05) higher FIS score in control group compared with intervention group	Robotic devices can help manage dental anxiety successfully
Abbasi et al. [23]	FIS: pre = 2.80, post = 2.50	NA	Good	NA	NA	Significant decrease in heart rate and FIS values	Mobile app found to be effective
Huang et al. [24]	Effective in reducing dental anxiety	NA	Effective	Reliable	12 months	The SUS value for WADA is 72.25, exceeding the average value	Effective for reducing treatment risks
Al Kheraif et al. [25]	GSR scores: pre = 1.79, post = 0.78; Venham anxiety and behaviour scale: pre = 2.96, post = 0.17; FBRS: with VR = 2.39, without VR = 3.14	10 patients were non‐compliant	Good	NA	NA	Reduced anxiety and better behaviour	Virtual reality and artificial intelligence interventions proved to be an effective approach
Shetty et al. [26]	RMS‐PS exhibited a strong correlation (0.75) with FIS scores	NA	Strong correlation	Satisfaction rate for RMS‐DAS = 63.6%	NA	Strong correlation between RMS‐DAS with RMS‐PS (*r* = 0.73) and FIS (*r* = 0.76)	The RMS‐DAS is a dependable and valid instrument for assessing dental anxiety

Abbreviations: BMP, behaviour management problems; DAS, digital anxiety scale; FBRS, Frankl Behaviour Rating Scale; FIS, Facial Image Scale; GSR, galvanic skin response; MDAS, Modified Dental Anxiety Scale; NA, not available; PS, pictorial scale; SUS, system usability scale.

### 3.5. Exploratory Quantitative Analysis

**Figure 2:**
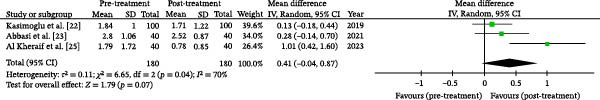
Forest plot of the impact of AI‐based interventions on dental anxiety.

**Figure 3:**
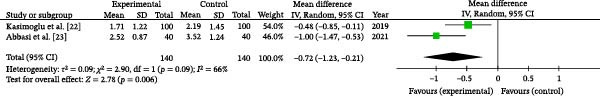
Forest plot for the post‐interventional impact of AI‐based interventions compared with the control group.

Quantitative pooling of study findings was feasible for a limited number of studies that provided numeric outcome data for pooling. Data were able to be pooled pre–post for three studies and post‐intervention (versus control condition) for two studies. Quantitative synthesis was considered exploratory due to the small number of studies contributing to the pooled dataset and clinical heterogeneity of study designs. Exploratory pooled estimates suggest that there may be some differential change in anxiety outcomes after intervention exposure. There was substantial heterogeneity in these pooled analyses, and the results are likely suggestive and not conclusive of treatment effects. Figures 2 and 3 are exploratory analyses and not confirmatory meta‐analysis results.

### 3.6. Methodological Quality Assessment

The methodological quality of included studies was evaluated with the MMAT. Each of the two RCTs met most of the appraisal criteria. Some concerns remained regarding the blinding of outcome assessment. All non‐randomised studies met several methodological criteria appropriate to their study designs. Given the overall heterogeneity among studies in design, intervention type, comparator structure and outcome measurement, the quality of evidence was sufficient to conclude that findings should be interpreted with caution, rather than that they were at low risk of bias.

## 4. Discussion

This systematic review collected the existing data on AI‐driven and digital interventions for dealing with assessment, monitoring and management of dental anxiety. Findings from the studies suggest that certain technology‐assisted approaches may help alleviate anxiety‐related symptoms, foster positive behavioural reactions, and enhance aspects of patient experience within the dental setting. Limitations of this evidence include a small number of studies that are methodologically and conceptually diverse. Studies were inconsistent in terms of study design, targeted age group, type of intervention, comparator and anxiety outcome assessment, which limits the strength of conclusions.

A major methodological limitation across studies identified by this review was an inconsistent definition of the key term ‘artificial intelligence’. Not all technologies evaluated here were representative of AI‐based therapies. ML and algorithm‐based assessment tools were often compared to broader digital technologies like smartphone applications, computerised behavioural programmes/interventions, VR, robotic platforms or digitalised anxiety scales. For this reason, the results of this review should not be considered reflective of one collective category of AI treatments. This review aimed to summarise available evidence specific to AI‐enabled and digital technology‐assisted tools that were applied to the assessment, prediction, monitoring, behaviour management or reduction of dental anxiety. This is an important distinction because assessment or predictive tools are not interpretable as anxiety‐reducing interventions administered during dental treatment.

Interventions based on these technologies could theoretically lessen dental anxiety through several mechanisms. Digital and AI‐assisted modalities could enhance predictability and sense of control, which influence anxiety related to medical procedures. Interactive tools, cognitive–behavioural programmes, and VR could serve as distraction and emotional coping tools, which could reduce preoperative fear and behavioural expression of distress. Robotics, biofeedback‐based technology, or app‐mediated communication could increase engagement, familiarity and sense of connection particularly with children or anxiety‐prone patients who may thrive on caring interactions. Physiological monitoring and feedback technology could theoretically allow for more individualised moment‐to‐moment anxiety management. This has not been explored in any well‐designed study [27–33].

Narrative results from the included studies were generally positive. C‐CBT was associated with reduced dental anxiety and avoidance behaviour. Robotic support appeared to improve child behaviour during treatment, and some mobile/VR‐linked interventions were associated with decreased anxiety scores or increased cooperation. Many studies did not necessarily attempt to directly test the efficacy of a given treatment. Some studies focused on scale validation, usability/participatory development and anxiety‐focused assessment (with no intervention) [8, 15, 34]. This is the reason that the overall findings are considered preliminary.

The quantitative results derived from our exploratory pooling were based on very limited data. Only a handful of studies reported countable outcomes eligible for pooling. There was marked clinical heterogeneity between studies both in terms of intervention provided as well as populations targeted. Our pooled estimates trend towards a benefit and our quantitative findings do not conclusively show that AI‐enabled or assisted interventions are effective at reducing dental anxiety across settings [35].

Despite these limitations, this study has the potential to make clinical contributions. The nature of dental anxiety management is complex and multifaceted. Many anxious patients respond to calming procedures that emphasise comfort, communication, engagement and procedural predictability. Technology‐based distraction modalities could serve as one tool among many supplemental aids. This works alongside traditional behaviour guidance techniques to manage anxiety for paediatric patients, special care patients and patients who request digital interventions. Technological distraction interventions should not replace evidence‐based anxiety management techniques. These are behaviour guidance, communication skills training, CBT or pharmacological interventions when indicated but should currently be considered investigational adjuncts in need of additional description of utility.

There are also several important negative limitations and concerns to be highlighted. Several positive findings described interventions that cannot be distinctly categorised as AI systems by our definition, creating conceptual ambiguity. There was heterogeneity of tools used to measure anxiety (i.e., MDAS, FIS, FBRS, GSR‐based tool, Venham scales, CFSS‐DS and RMS‐based anxiety scales). The evidence included both randomised and non‐randomised studies (retrospective studies, comparative studies, participatory design studies and cross‐sectional studies), which carry with them different levels of inferential power. Fourth, several studies had small sample sizes, short durations of follow‐up, and were unblinded. Last, the implementation of AI‐enabled dental technology at scale incurs additional concerns. These include data privacy, informed consent, algorithmic interpretable ML, fairness, cybersecurity, accessibility and cost.

Measures used in psychiatric anxiety literature (i.e., Hamilton Anxiety Rating Scale, HAM‐A; Generalised Anxiety Disorder‐7 scale, GAD‐7; State–Trait Anxiety Inventory, STAI; Beck Anxiety Inventory, BAI; Hospital Anxiety and Depression Scale, HADS) were not used across studies. Dental chairside‐feasible measures such as the MDAS, FIS, FBRS, Venham scales, CFSS‐DS, GSR‐based measures of arousal, and RMS‐based measures were more commonly used across studies. These measures have been perceived to be more clinically sensitive to dental settings for use with paediatric, procedural, and special‐care populations.

A further limitation is the conceptual heterogeneity of the included technologies. All studies included were related to dental anxiety, but technologies varied widely in their intended purpose. Some studies were conducted on technologies designed to be active anxiety reduction or behaviour‐management interventions. Many other studies were designed primarily for anxiety assessment, prediction, scale validation or usability testing purposes. These findings should not be interpreted as evidence that all AI‐enable or digital tools are effective in dental anxiety reduction. Evidence suggests that selected digital and AI‐enabled approaches can be used in support of dental anxiety assessment and management but that their therapeutic benefit is currently unsubstantiated.

The present review has also highlighted a methodological issue, which we feel is of broader relevance. There is a critical need for future studies within this domain to clearly state what AI is and what is not AI. When reporting on ML approaches, adaptive predictive algorithms, intelligent virtual humans, automated biofeedback and digitally based interventions that do not utilise AI, researchers should not automatically cluster these together when pooling data for analysis unless there is some conceptual rationale for doing so. The classification of interventions can be improved. This would enable a better synthesis of evidence and clinical interpretation of findings. Future studies should aim to utilise randomised controlled designs where possible. They should have well‐defined comparator groups, standardised and clinically meaningful anxiety measures and include long‐term follow‐up to determine whether effects of the interventions are maintained. Studies should also assess the feasibility of implementing AI into practice. These include patient acceptability, ethical issues and cost benefits within real world dental environments.

Additional research considerations involve how such technologies could interface with existing clinical workflows. AI‐enabled screening devices could be used to detect highly anxious patients before their scheduled appointment. Digital behavioural support or VR‐based distraction could be implemented into the workflow for select patients as part of chairside care. Biofeedback‐enabled technologies could potentially be used in the future to monitor anxious or special needs patients in real‐time. Much stronger evidence would be needed before any of these frameworks could be widely suggested. More research is necessary to identify what modalities are effective and which work best for patients as per clinical situations. This technology will provide benefits outside of traditional care.

Taken together, our findings suggest that AI‐based and digitally mediated approaches are likely novel yet unproven treatments for dental anxiety. The existing data suggests a more cautious response is warranted. Well‐designed and clinically standardised studies are required prior to implementing digital dentistry tools as evidence‐based treatments for dental anxiety.

## 5. Conclusions

AI‐enabled and digital technology‐assisted tools may aid in dental anxiety assessment, monitoring, prediction, behaviour management and anxiety reduction. The evidence base is limited by small numbers of studies and conceptual heterogeneity of technology platforms. There are also varied purposes of intervention and methodological limitations. The included technology did not always reflect AI‐based therapeutic approaches, and numerous technologies were broad digital programs or assessment only. Future technologies cannot be considered established evidence‐based approaches to treat dental anxiety until the limitations highlighted above are addressed. AI‐based digital therapies being studied should report what specific AI or digital aspect is being implemented, whether the tool is being used to assess anxiety or reduce anxiety, use standardised dental anxiety outcome measures and report on well‐defined comparator groups with appropriate follow‐up.

## Funding

The authors extend their appreciation to the Deanship of Research and Graduate Studies at King Khalid University for funding this work through the Review Article Project under the Grant RA.KKU/7/45.

## Conflicts of Interest

The authors declare no conflicts of interest.

## Supporting Information

Additional supporting information can be found online in the Supporting Information section.

## Supporting information


**Supporting Information** Table S1: Worldwide web addresses used for literature research.

## Data Availability

The data that support the findings of this study are available from the corresponding author upon reasonable request.
