# Supplementary material for: Systematic Review and Exploratory Meta‐Analysis of AI‐Enabled and Digital Technology‐Assisted Interventions for Dental Anxiety During Dental Treatment
Source: Depress Anxiety. 2026 Jul 2;2026:8850179. doi: 10.1155/da/8850179 (PMC13324240; doi:10.1155/da/8850179)
Supplement: Supplementary file 1 — Supporting Information Table S1: Worldwide web addresses used for literature research. [file DA-2026-8850179-s001.docx]

| 1 | PubMed | https://pubmed.ncbi.nlm.nih.gov/ |
| --- | --- | --- |
| 2 | Scopus | https://www.scopus.com/ |
| 3 | ScienceDirect | https://www.sciencedirect.com/ |
| 4 | Google Scholar | https://scholar.google.com/ |
| 5 | Cochrane Library | https://www.cochranelibrary.com/ |

Supplementary Table S1: Worldwide web addresses used for literature research.
